# Autopsy pathology should become a recognised subspecialty

**DOI:** 10.1007/s00428-008-0595-8

**Published:** 2008-03-06

**Authors:** Jan G. van den Tweel

**Affiliations:** Department of Pathology H4 312, University Medical Center Utrecht, Heidelberglaan 100, 3584 CX Utrecht, The Netherlands

**Keywords:** Intensive Care Medicine, Residency Training Programme, Request Form, Autopsy Rate, Clinical Colleague

Dear Editor,

To say that autopsy rates are declining is like kicking an open door. For more than three decades, hundreds of articles have been written to discuss this phenomenon [6]. No matter the background of the authors, clinicians and pathologists alike regret this decline and are of the opinion that this situation should be reversed in order to assure high quality medical practice [3–5, 7, 9]. However, all these papers have not had the desired effect of reversing this process. It appears that these papers served mainly to benefit the curriculum vitae of the respective authors and their list of citations. In strong contrast with this supposed lack of interest is the fact that a PubMed search for the term “autopsy” over the years 2000–2006 shows more than 10,000 links, indicating that the autopsy is still an important technique to study (mechanisms of) disease.

The reasons for this decline are well known and extensively described [3]. Relatives now are more hesitant than in the past and refuse approval for an autopsy more frequently; or, perhaps, we should say that relatives in the past were as hesitant as relatives are at present but were less likely to contradict the then-well-known (and almighty) doctor. This independence has to be accepted as a fact, there is no reason to believe that this tide will change.

Clinicians share some of the blame. Today, they are, perhaps, less persistent and even less interested in pursuing an autopsy because contacts with recently bereaved families are difficult, and many clinicians believe that modern imaging techniques supply them with enough critical information. Moreover, in today’s specialty and clinic orientated practice, the clinicians have less personal contacts with the patient and the relatives, and the chance that a patient dies in the presence of his own doctor is not what it was in the past. This situation will not change in the near future. Therefore, this looser relationship has to be accepted as a fait accompli, and no drastic changes can be foreseen that will influence the autopsy numbers in this respect.

Finally, the various ‘medical authorities’ are also not very helpful. Requirements for specified numbers of autopsies for certification of hospitals and for residency training programmes were abolished long ago, often followed by the withdrawal of funding to perform autopsies.

So what are we left with? Do we have to accept the situation as it is, or is there something that we, as pathologists, can do? It is strange that pathologists, although we must share responsibility for the waning of the autopsy, often are left in the shadow. Sometimes, long delays in the reporting of autopsy findings is mentioned as a cause of clinical dissatisfaction, but the professional competence of pathologists is almost never an issue. Yet a lot can be gained here. What are the facts?

- Many pathologists are not interested in autopsies and/or see them as a burden.
- Many pathologists lack sufficient clinicopathological background to adequately answer the questions of their clinical colleagues.
- Reports are often so delayed that the critical features of the case have been lost from memory, and interest therefore, is low.
- Reports often do not contain the relevant answers.
- In some occasions, the reported results are evidently wrong.

Let us examine these facts one by one.

## Many pathologists are not interested in autopsies

There is nothing wrong with the fact that some, or even many, pathologists have little direct interest. We accept that we have among our colleagues dermatopathologists, GI pathologists, etc. But for some reason, there is a widely held perception that every pathologist should be able to perform, or at least supervise, autopsies “to equally share the burden”, as is often said. This is a very strange and illogical perception. Nobody expects a general pathologist to diagnose a difficult myeloproliferative disorder or an unusual neurodegenerative disease or recognize a Sanderson’s polster, when they see one. But we nevertheless expect him/her to perform with aplomb an autopsy on a complicated intensive care unit (ICU) patient with multiorgan disease. The same problem is also seen at resident level. The UK already offers pathology training programmes without autopsy training. At a recent course for first year pathology residents in the Netherlands, I asked the 25 residents how many of them would choose such a training if offered. Five answered positively and six indicated that they would prefer never to do autopsies as a pathologist. Worse is the situation when pathologists openly put the value of autopsies up for debate. Unfortunately, this happens with increasing frequency.

## Many pathologists lack sufficient pathophysiological knowledge

Performing autopsies not only requires a solid basis in the field of general pathology but also in the common problems of clinical medicine and in basic anatomy and physiology. The pathophysiology of the circulatory, respiratory and renal system, in addition to that of the clotting system, should be well known. Familiarity with ICU complications and their treatment is mandatory for adequate interpretation of what may be encountered in many autopsy cases. Also, the interpretation of the pathology of shock versus post mortem changes requires special training. We cannot expect that every pathologist has the time and interest to keep his competencies updated in such a broad and complex field, certainly if he/she, with the declining autopsy rates, will only occasionally be responsible for an autopsy.

## Reports are often delayed

The fact that autopsies are often reported after many weeks or even months (and sometimes not at all) is well known and constitutes a complaint in various articles discussing autopsy decline. The responsible clinician has, in the meantime, often moved to another rotation or has forgotten the details of the disease history and has also sent the final report about the patient to the GP of the patient; thus, the case is ‘closed’ in his/her mind. This type of disconnect certainly is a source for irritation and will contribute to reluctance in asking another autopsy approval.

*Many autopsy reports do not contain relevant answers* to the questions raised during life. It must be said that the autopsy request forms often contain marginal and/or incomplete information [2]. However, the questions raised should be answered and in an epilogue the morphological findings should be interpreted in the clinical context and vice versa. Only then the patient’s file can be closed with confidence.

## In some instances, the reports provide wrong information

These types of errors can be due to misinterpretation of findings, often because of lack of detailed knowledge of the clinical course leading up to death, coupled with lack of insight in pathophysiological phenomena or due to hurry or even neglect [2]. The circumstances surrounding Dr. Shipman’s cases are a good example of this situation, where deaths were reported as cardiac events without sufficient evidence for such a diagnosis [8]. Similar situations might happen everywhere. This type of publicity, certainly when combined with tissue retrieval problems, will most certainly influence the attitude towards autopsies of the general public and medical professionals.

These facts taken together raise real concern as to the contribution of ‘Pathology’ and pathologists in the decline in autopsy rates and, in effect, cry out for another approach by our profession to the performance of autopsies. Moreover, we cannot close our eyes to such new approaches as limited autopsies, CT and MRI procedures followed by needle biopsies and so on [1, 3]. Certainly, in the light of the declining autopsy rates, where it is difficult to acquire sufficient experience in performing, interpreting and reporting autopsies, it is necessary that the pathologists who perform autopsies are enthusiastic, interested, competent and respected for their knowledge in this field. Only then are they good sparring partners for the clinicians and good teachers for our residents.

The only way to achieve this goal is to accept Clinical Autopsy Pathology as a recognised subspecialty within our profession, in the same way as other subspecialties. A period of additional training in intensive care medicine would be desirable—to learn the language of the intensive care unit which, because of the critical condition of many ICU patients, is the source of a significant number of autopsies and certainly the most complicated ones. Or possibly, there is even a case for incorporating a year of clinical experience in Medicine and Surgery into the training of a Clinical Autopsy Pathologist, an experience that surely contributed to the high level of competence that many of our illustrious predecessors displayed in autopsy pathology. Of course, there should be room for subspecialists who will usually perform their own autopsies, e.g. paediatric pathologists and neuropathologists.

In conclusion, the decline in autopsy rates is a progressive one. It is time for us, the pathologists, to finally do something constructive other than to weep and wail. We have to bring our best men and women in the frontline and respect their work as highly as any other recognised subspecialty. It is that or the end of the autopsy. What if our attempts do not work, and the decline continues? In that case, we have already anticipated some of the measures that must be adopted anyway. There is no way to escape the future; it is coming. But we can reshape it by acting now. I, therefore, sincerely hope that this letter will not prove to be just an addition to my list of publications.

## References

[1] Benbow EW, Roberts ISD (2003). The autopsy: complete or not complete. Histopathology.

[2] Bove KE, Iery C, Autopsy Committee of the College of American Pathologists (2002). The role of autopsy in medical malpractice cases. II. Controversy to autopsy performance and reporting. Arch Pathol Lab Med.

[3] Burton JL, Underwood J (2007). Clinical, educational, and epidemiological value of autopsy. Lancet.

[4] Eresi M, Ansorge O (2007). Autopsy: not dead. Lancet.

[5] Hull MJ, Nazarian RM, Wheeler AE, Black-Schaffer WS, Mark EJ (2007). Resident physician opinions on autopsy importance and procurement. Hum Pathol.

[6] Roberts WC (1978). The autopsy: its decline and a suggestion for its revival. N Engl J Med.

[7] Roulson J, Benbow EW, Hasleton PS (2005). Discrepancies between clinical and autopsy diagnosis and the value of post mortem histology. Histopathology.

[8] HSMO (2003). The Shipman Inquiry, third report: death certification and investigation of death by coroners.Cm 5854.

[9] Zarbo RJ, Baker PB, Howanitz PJ (1999). The autopsy as a performance measurement tool–diagnostic discrepancies and unresolved clinical questions: a College of American Pathologists Q-Probes study of 2479 autopsies from 248 institutions. Arch Pathol Lab Med.

